# Research on Structural Optimization and Excitation Control Method Using a Two-Dimensional OWPT System for Capsule Robots Based on Non-Equivalent Coils

**DOI:** 10.3390/mi15121510

**Published:** 2024-12-19

**Authors:** Wenwei Li, Pingping Jiang, Zhiwu Wang, Guozheng Yan

**Affiliations:** 1 School of Electronic Information and Electrical Engineering, Shanghai Jiao Tong University, Shanghai 200240, China; 1737631240lww@gmail.com (W.L.); zwwang@sjtu.edu.cn (Z.W.); 2 School of Biomedical Engineering, Shanghai Jiao Tong University, Shanghai 200240, China; gzhyan@sjtu.edu.cn

**Keywords:** capsule robots, Helmholtz coil, saddle coil pairs, wireless power transfer, omnidirectional magnetic field, S-S topology

## Abstract

The rapid development of wireless power transfer (WPT) technology has provided new avenues for supplying continuous and stable power to capsule robots. In this article, we propose a two-dimensional omnidirectional wireless power transfer (OWPT) system, which enables power to be transmitted effectively in multiple spatial directions. This system features a three-dimensional transmitting structure with a Helmholtz coil and saddle coil pairs, combined with a one-dimensional receiving structure. This design provides sufficient internal space, accommodating patients of various body types. Based on the magnetic field calculation and finite element analysis, the saddle coil structure is optimized to enhance magnetic field uniformity; to achieve a two-dimensional rotating magnetic field, a phase difference control method for the excitation signal is developed through the analysis of circuit topology and quantitative synthesis of non-equivalent magnetic field vectors. Finally, an experimental prototype is built, and the experimental results show that the one-dimensional transmitting coil achieves a minimum received voltage stability of 94.5% across different positions. When the three-dimensional transmitting coils operate together, a two-dimensional rotating magnetic field in the plane is achieved at the origin, providing a minimum received power of 550 mW with a voltage fluctuation rate of 7.68%.

## 1. Introduction

### 1.1. Research Background 

The rapid advancement of wireless power transfer (WPT) technology has led to its widespread application in various fields, including electric vehicles, autonomous underwater vehicles, industrial automation equipment, IoT devices, implantable medical devices, consumer electronics, and portable electronic devices [1,2,3,4]. WPT enables power transfer without physical contact.

In particular, implantable medical devices such as capsule robots (CRs) integrate power-consuming components like actuators and cameras, resulting in power consumption typically reaching hundreds of milliwatts [5,6,7,8,9,10]. Power supply in CRs heavily relies on onboard batteries; however, battery capacity is proportional to volume, which makes it challenging to achieve long-term stable power supply while also miniaturizing the CRs. Replacing onboard batteries with wireless power transfer technology can effectively reduce the load on CRs, allowing for a lighter design and long-term stable power supply. Currently, WPT based on inductive coupling principles is considered the most suitable power transfer method for CRs [6], where the power transfer path runs from the external transmitting (Tx) coil to the internal receiving (Rx) coil.

However, several key challenges remain in implementing wireless power transfer. First, most WPT systems for CRs employ one-dimensional Tx coils and three-dimensional Rx coils, which unavoidably increases the CR’s spatial volume. Second, the movement of CRs within the human body is irregular, with frequent changes in its position and, sometimes, its angle, necessitating a uniform magnetic field that can cover the workspace. Third, from the perspective of patient usability, the setup of the transmitting structure requires sufficient internal space. Therefore, conventional WPT systems are not suitable for these applications.

### 1.2. Related Research Work

To enhance the freedom of wireless power transfer for implantable medical devices, researchers have devoted considerable effort to optimizing coil structure design, excitation signal, and other factors to achieve multi-dimensional power transfer. Some studies have employed a one-dimensional Tx coil and a three-dimensional Rx coil. A few of these studies [11,12,13,14,15,16,17] have focused on one-dimensional Tx coils, including the Helmholtz coil, single solenoid coil, double solenoid coils, and planar spiral coil. These structures can produce uniform magnetic fields and deliver hundreds of milliwatts of power but do not achieve omnidirectional magnetic fields. Research on three-dimensional Rx coils [18,19,20,21,22,23] is often complicated by issues such as complex internal circuitry and significant spatial demands, which can prevent the CR from carrying enough functional modules and increase its movement load, hampering long-term stable operation. There are also studies that have focused on three-coil systems. For instance, the spherical receiver coil proposed by Basir et al. demonstrates excellent performance in power transfer under angular misalignment within a three-coil system [24]. However, the requirement for close proximity between the transmitting and receiving coils is challenging to achieve in practical applications, and there remains significant potential for improving resistance to positional shifts.

To enhance the integration of CRs and incorporate more functional modules, WPT systems applied to CRs need to focus on solutions with multi-dimensional transmitting coils and one-dimensional receiving coils to provide a uniform magnetic field while also increasing the omnidirectional capability of the magnetic field. Omnidirectional wireless power transfer (OWPT) technology has gradually become a research hotspot in the field of wireless power transfer [25,26,27,28] thanks to its strong anti-interference capability, wide transfer orientation, and excellent adaptability to attitude shifts, significantly expanding the application scenarios of implantable medical devices [29,30,31]. 

In terms of implementation, omnidirectional magnetic fields can be classified into targeting magnetic fields directed at the receiving part [32,33,34] and rotating magnetic fields [33,35,36,37,38,39,40,41]. Targeting magnetic fields typically require additional attitude detection modules at the receiving part, which is not suitable for the miniaturization of CR systems. For example, Ye et al. proposed a WPT system using three-dimensional orthogonal circular coils, but it requires phase adjustment based on the receiving part’s attitude, and the transmitting part can be improved for more internal space. For the regulation of rotating magnetic fields, structures composed of multiple rectangular coils or saddle coils can theoretically be naturally decoupled and are widely used in two-dimensional omnidirectional systems [36,38,39,40,41]. For instance, some researchers have proposed a WPT system consisting solely of saddle transmitting coils, which generates a two-dimensional rotating magnetic field through phase modulation of the excitation currents; however, the proposed coils fail to sufficiently account for the uniformity of the magnetic field. Additionally, several new coil structures have been proposed [33,35,37], such as three-pole planar coils and mesh planar transmitting coils, which, despite having good omnidirectional transfer performance, require the transmitting and receiving structures to be in close proximity; the methods employed, such as current amplitude modulation, are also quite complex. It is worth noting that the coils mentioned above are either structurally identical or require that the magnetic field components generated in each direction be of equal magnitude. However, there is a lack of sufficient analysis for scenarios where the three-dimensional components are not precisely equal in spatial configuration.

### 1.3. Major Contributions

To overcome the above limitations, this article proposes a non-equivalent coil OWPT system using a Helmholtz coil and saddle coil pairs to power CRs in cylindrical spaces. We also propose a phase control method for synthesizing spatial magnetic field vectors, ensuring the realization of a two-dimensional rotating magnetic field. A system prototype is built for experimentation. The proposed system has the following main advantages:

1. A three-phase cylindrical open structure of Helmholtz coils combined with saddle coil pairs is proposed for the treatment needs of patients with different body types.
2. Based on the uniformity of the spatial magnetic field, the key structural parameters of the saddle coil pair have been optimized, which means that the receiving mechanism has a high positional tolerance, ensuring sufficient power can be picked up during angle alignment.
3. For the non-equivalent magnetic field in three-dimensional space, the phase difference of the excitation currents is optimized, achieving a two-dimensional rotating magnetic field, with excellent tolerance to attitude shifts at the center.
4. Only one DC power supply is required, saving resources.

### 1.4. Article Organization

The rest of this article is organized as follows: Section 2 introduces the proposed OWPT system and the design requirements for CRs. In Section 3, we describe a three-dimensional power transfer structure consisting of a Helmholtz coil and saddle coil pairs, and we determine the geometric parameters of the coils and the operating frequency of the system while optimizing the magnetic field uniformity through magnetic field analysis and finite element simulation. In Section 4, we introduce the equivalent circuit model of the system, quantitatively analyze the relationship of non-equivalent spatial magnetic field vector synthesis, and present a method for regulating the phase difference of excitation currents. In Section 5, we describe a prototype of the proposed OWPT system and verify the correctness of the theoretical analysis through simulation and experimentation. Finally, the discussion and conclusions are provided in Section 6.

## 2. Overview of the Proposed OWPT System 

### 2.1. Framework of the Proposed OWPT System

**Figure 1:**
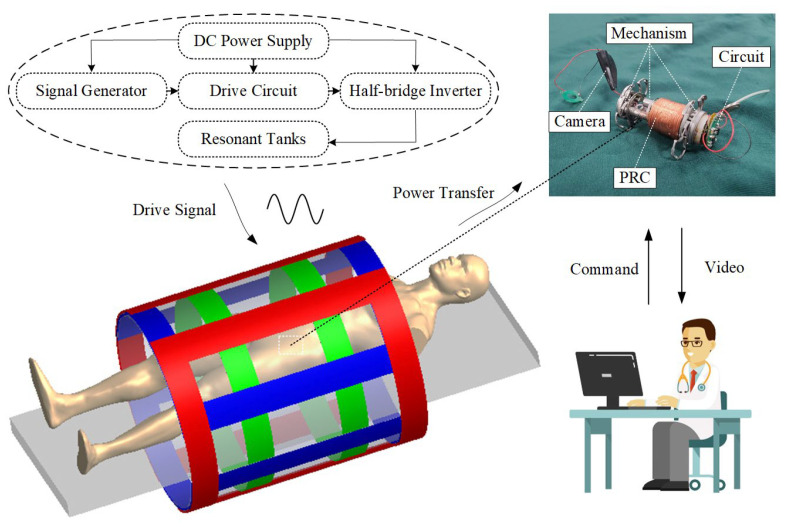
Framework of the proposed OWPT system.

In Figure 1, a framework of the proposed OWPT system is shown. In this OWPT system, the power transfer mechanism comprises three pairs of transmitting coils wound on a PVC cylinder, and the power receiving mechanism is a one-dimensional coil. The power transmitting part generates three-phase control signals from a signal generator, which control three-phase half-bridge inverters via a drive circuit. Each sinusoidal alternating signal is generated by each series resonant tank to excite an alternating magnetic field in the transmitting coils, which is vectorially synthesized in space. The power is picked up by the receiving coils of the CR in the body of the power receiving part. Through a full-bridge rectifier circuit and a linear voltage regulator, the CR will receive a stable DC voltage to be used to drive the motion mechanism to ensure continuous motion and normal diagnosis in the intestine.

### 2.2. Structure of Transmitting and Receiving Coils

**Figure 2:**
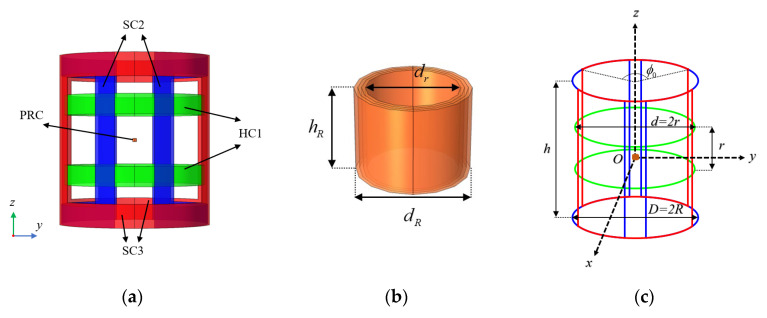
3D model (**a**,**b**) and simplified wireframe (**c**) of the coils.

The A-phase coil is a Helmholtz coil, which consists of two sets of identical circular coils connected in series and is denoted as HC1 (green); the B- and C-phase coils are saddle coil pairs, which consist of two sets of identical saddle coils connected in series and are denoted as SC2 (blue) and SC3 (red), respectively; and the power receiving mechanism, denoted as PRC, is a densely packed coil embedded in the CR and wound in three layers. Their 3D model and simplified line structure are shown in Figure 2a–c. The diameter of HC1 is d and the spacing between the two sets of circular coils is its radius r. SC2 and SC3 have the same parameters, with diameter D and height h. Their length–radius ratios are defined as λ=h/R, and the corresponding center angle of the arc is $\phi_{0}$. The PRC is wound on a plastic cylinder with an inner diameter of $d_{r}$, an outer diameter of $d_{R}$, and a height of $h_{R}$. It is wound in three layers from the inside out, with each layer having the same number of turns $N_{r}$, resulting in a total number of turns of $N_{R}=N_{r}\times 3$ for the PRC. The number of turns of the transmitting coils in each phase are N×2. Among them, the A-phase coil HC1 mainly generates the magnetic field in the *z*-axis direction, the B-phase coil generates the magnetic field in the *y*-axis direction, and the C-phase coil generates the magnetic field in the *x*-axis direction. HC1 and SC2/SC3 are all designed to occupy little space, and the patient can lie in them comfortably while the CR performs the task. 

## 3. Magnetic Field Modeling and Transmitting Coil Optimization

### 3.1. Magnetic Field Modeling of the Transmitting Coils

**Figure 3:**
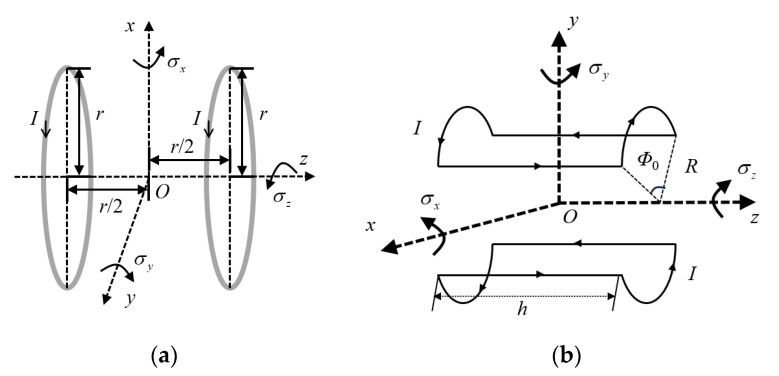
Excitation current definitions of (**a**) the Helmholtz coil and (**b**) the saddle coil.

To facilitate the analysis of the spatial magnetic field, Figure 3a,b shows the simplified models of the Helmholtz coil HC1 (a) and the saddle coil pair SC2 (b), respectively, with the defining direction of the excitation currents. $\sigma_{x}$ is the angle of rotation of the PRC around the *x*-axis, $\sigma_{y}$ is the angle of rotation of the PRC around the *y*-axis, and $\sigma_{z}$ is the angle of rotation of the PRC around the *z*-axis. 

Based on Biot-Savart’s Law, the magnetic field strength $H_{L}$ generated by the current-carrying conductor **L** at any point in space can be expressed as

(1) $$H_{L}=\frac{I}{4\pi }\int \frac{dL\times r}{r^{3}}$$

where I is the input current and r is the distance from the current element IdL to the point.

Assuming the coordinate system is centered at the geometric center of an N-turn circular coil, the magnitude of the component of the magnetic field strength in the *z*-axis direction generated by the N-turn circular coil at any point (*x*, *y*, *z*) in space can be expressed as

(2) $$\left\{\begin{array}{l} H_{z}=H_{z0}\int_{0}^{2\pi }\left[\frac{r^{3}-yr^{2}\sin\theta_{r}-xr^{2}\cos\theta_{r}}{{\left(x^{2}+y^{2}+z^{2}+r^{2}-2xr\cos\theta_{r}-2yr\sin\theta_{r}\right)}^{3/2}}\right]d\theta_{r}\\ H_{z0}=\frac{NI}{4\pi r} \end{array}\right.$$

where r is the radius of the coil and $\theta_{r}$ is the angle between a point on the coil and the *x*-axis in the positive direction. 

For the Helmholtz coil in Figure 3a, which is an N-turn circular coil placed symmetrically on both sides, the expression for the *z*-axis direction component of the magnetic field strength at any point (*x*, *y*, *z*) in space can be expressed as

(3) $$H_{z,HC1}=H_{z}\left(x,y,z+\frac{r}{2}\right)+H_{z}\left(x,y,z-\frac{r}{2}\right)$$

At the origin, the magnetic field strength generated by HC1 can be expressed as

(4) $$H_{z,HC1}\left(0,0,0\right)={\left(\frac{4}{5}\right)}^{3/2}\frac{NI_{A}}{r}$$

where $I_{A}$ is the current flowing through HC1.

For the saddle coil pair SC2 symmetrically placed with N turns on both sides in Figure 3b, based on the right-hand screw rule and structural symmetry, its magnetic field strength at the origin is the superposition of the magnetic field generated by each line segment, and this component only exists on the *y*-axis.

(5) $$H_{y,SC2}\left(0,0,0\right)=\frac{NI_{B}}{\pi R}\sin\frac{\phi_{0}}{2}\cdot \left(\frac{8R^{2}h}{{\left(h^{2}+4R^{2}\right)}^{3/2}}+\frac{2h}{{\left(h^{2}+4R^{2}\right)}^{1/2}}\right)$$

where $I_{B}$ is the current flowing through SC2. Symmetrically, the expression for the component of the magnetic field strength at the origin of SC3 in the *x*-axis can be expressed as

(6) $$H_{x,SC3}\left(0,0,0\right)=\frac{NI_{C}}{\pi R}\sin\frac{\phi_{0}}{2}\cdot \left(\frac{8R^{2}h}{{\left(h^{2}+4R^{2}\right)}^{3/2}}+\frac{2h}{{\left(h^{2}+4R^{2}\right)}^{1/2}}\right)$$

where $I_{C}$ is the current flowing through SC3.

The equivalent plane of HC1 is in the *xOy* plane, generating a *z*-axis magnetic field; the equivalent plane of SC2 is in the *xOz* plane, generating a *y*-axis magnetic field; and the equivalent plane of SC3 is in the *yOz* plane, generating an *x*-axis magnetic field. The working planes of HC1, SC2, and SC3 are mutually perpendicular. According to the superposition of the magnetic field vectors, the synthetic alternating magnetic field H produced by them at the origin can be expressed as

(7) $$H=H_{z,HC1}k+H_{y,SC2}j+H_{x,SC3}i$$

where i, j, and k are the unit vectors corresponding to the *x*, *y*, and *z* axes, respectively.

After determining the coil parameters, the magnitude of the synthetic magnetic field strength at the origin is solely related to the transmitting currents. While calculating the magnetic field strength at the origin is relatively straightforward, the expression for the magnetic field strength in regions away from the origin becomes complex and difficult to analyze. Therefore, this article employs the finite element analysis method to conduct a more detailed examination of the magnetic field and optimization of the coil parameters.

### 3.2. Saddle Coil Optimization Based on Finite Element Analysis

The Helmholtz coil HC1 has been shown to produce a wide uniform magnetic field at the center axis. For the saddle coil pairs SC2 and SC3, only SC2 is analyzed as an example (below) since they are similar in design. For a general saddle coil pair, there exists an optimal value of λ=4 and $\phi_{0}=120^{\circ }$ for the ratio of height to radius λ and the central angle $\phi_{0}$, which results in the highest uniformity of the magnetic field [42], but it takes into account the uniformity of the magnetic field only in a very small area around the origin. In practice, it is difficult to satisfy the ratio of height to radius for the saddle coil pair; λ=2.3 is selected in this article, and the corresponding theoretical optimal central angle is 126°. For implantable medical devices, the CR can be considered to be active in a range of 20 cm × 20 cm on the plane [19], and in order to find a relatively uniform magnetic field distribution in space, the magnetic field uniformity of the saddle coil pair is defined as

(8) $$\gamma ={\left(1-\frac{\left|H_{y,SC2}\left(x,y,z\right)-H_{y,SC2}\left(0,0,0\right)\right|}{H_{y,SC2}\left(0,0,0\right)}\right)}_{\min}\times 100\%$$

**Figure 4:**
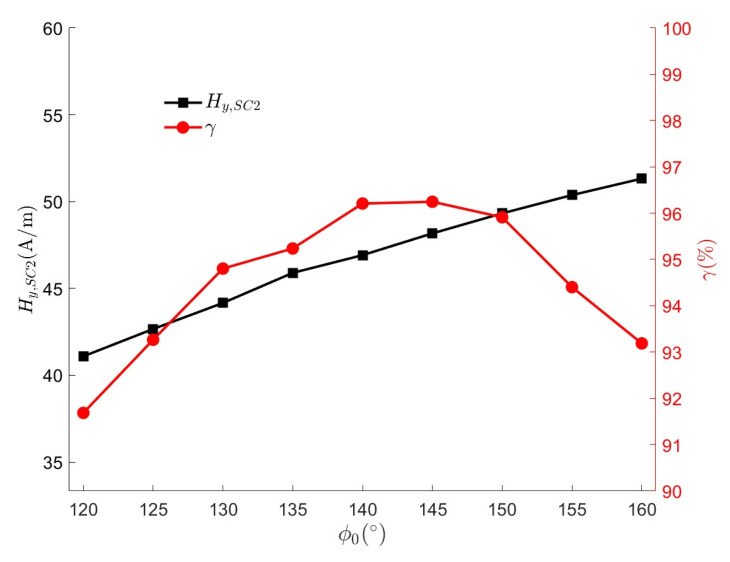
$H_{y,SC2}$ and γ at different $\phi_{0}$.

Without considering the effect of the width of the coil, magnetic field uniformity was calculated using COMSOL Multiphysics 6.0 finite element simulation software, and the spatial magnetic field close to the actual situation was obtained. In Figure 4, it can be seen that increasing the central angle leads to a larger magnetic field strength, which is caused by the increase in self-inductance with the effective length of the coil arc. The maximum magnetic field uniformity of 96.2% is achieved for the saddle coil pair at a central angle of 145°, which means that the saddle coil pair can provide a wide and uniform magnetic field distribution in a plane of 20 cm × 20 cm, and the reason for the decrease beyond this angle may be the edge effect of the magnetic field. Considering the abovementioned factors, a central angle of 150° was chosen to ensure sufficient magnetic field strength while maintaining a magnetic field uniformity of 95.9%.

For an OWPT system, the quality factor of the coil is very important as it is related to the power loss of the system and thus directly affects the transfer efficiency. The quality factor of the coil is defined as

(9) $$Q=\frac{\omega L_{B}}{R_{B}}$$

**Figure 5:**
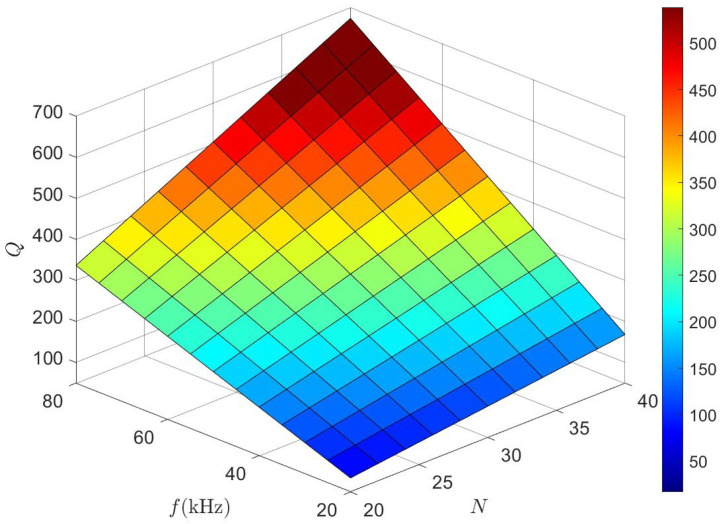
Quality factor of saddle coil pair versus frequency and number of turns.

**Figure 6:**
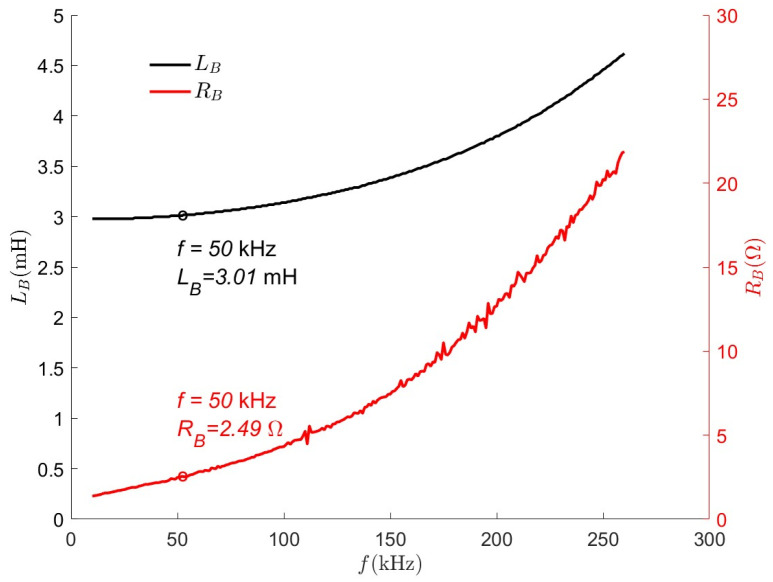
Self-inductance and internal resistance versus frequency.

The number of turns of the coil and the operating frequency affect the quality factor of the coil; a reasonable range of the quality factor is beneficial to enhance the magnetic field strength, and a quality factor that is too high may make it difficult to realize the withstand voltage of the compensation capacitor ($U_{C\max}=V_{DC}Q$). For the proposed OWPT system, the effect of operating frequency on coil self-inductance and coil internal resistance needs to be considered. According to Figure 5, increasing the number of turns and operating frequency can increase the quality factor. However, the quality factor is not an uncontrolled increase; considering the abovementioned factors, it was initially determined that the operating frequency range is 45–55 kHz, and the number of turns of the coil should be 28–30 turns, corresponding to a quality factor of 265 to 370. Considering the power demand, the number of turns of the coil N was set to 30. In order to further determine the operating frequency, Figure 6 shows the trend of coil self-inductance and internal resistance at different operating frequencies in the simulation. As the operating frequency increases, both the self-inductance and the resistance of the coil rise sharply. The increase in resistance leads to greater energy loss, which also raises the demand for the power supply’s driving capability. Additionally, frequency affects the electromagnetic safety of the system. Finally, the operating frequency is selected as 50 kHz, with sufficient power supply and relatively low loss.

## 4. Circuit Analysis and Derivation of Phase Difference Theory

### 4.1. Three-Phase Circuit Topology Analysis

**Figure 7:**
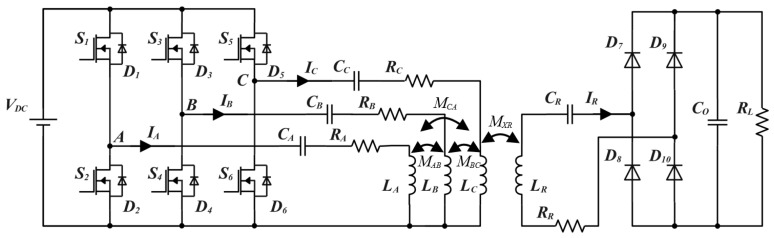
Three-phase S-S topology.

Figure 7 illustrates the topology of the three-phase OWPT system, where the coil of each phase is driven by a set of independent half-bridge inverters; S1~S6 represent the switches of the three-phase half-bridge inverter circuits, and a set of DC power supply is shared by all inverters, which conserves resources. $L_{A}$, $L_{B}$, $L_{C}$, and $L_{R}$ represent the self-inductance of the transmitting and receiving coils, and $C_{A}$, $C_{B}$, $C_{C}$, and $C_{R}$ represent the series compensation capacitors of the transmitting and receiving coils. $R_{A}$, $R_{B}$, $R_{C}$, and $R_{R}$ represent the equivalent series resistors of the transmitting and receiving coils, $M_{AB}$, $M_{BC}$, and $M_{CA}$ represent the mutual inductance between the transmitting coils, and $M_{XR}\left(X=A,B,C\right)$ represent the mutual inductance between the transmitting and receiving coils.

**Figure 8:**
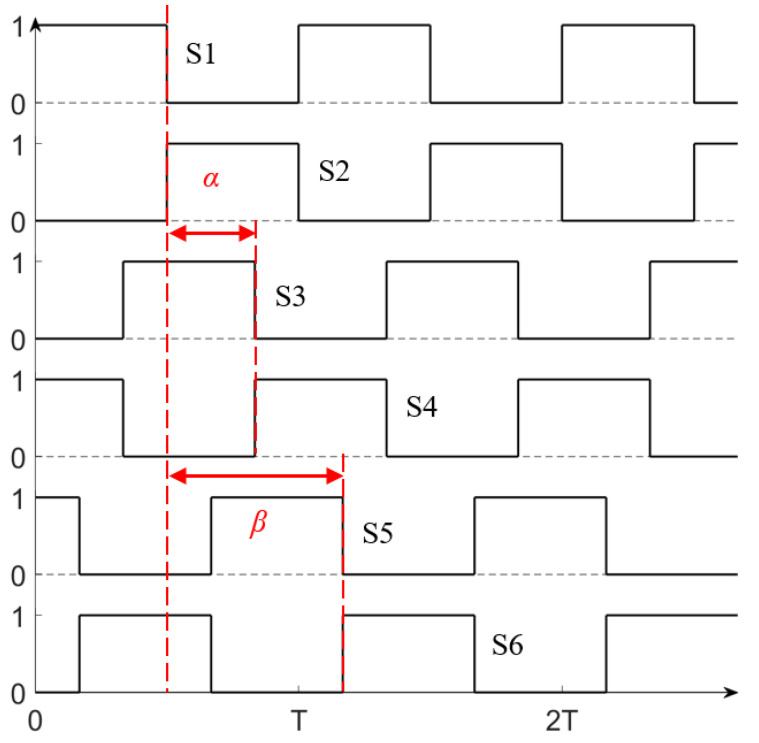
Timing diagram of excitation signal.

The outputs of the three-phase half-bridge inverter are independent of each other, and the upper and lower switching tubes of each group of half-bridges turn on or turn off in sequence. The excitation signals of each group of half-bridges have an identical duty cycle and dead time; their waveforms are shown in Figure 8. The output voltages of the three-phase inverter are $U_{A}$, $U_{B}$, and $U_{C}$. The voltages of the B-phase and C-phase lag behind the A-phase by α and β, respectively, which is called the phase difference of excitation signals. Based on the fundamental harmonic approximation method, ${\overset{•}{U}}_{X}\left(X=A,B,C\right)$ can be expressed as

(10) $$\left\{\begin{array}{l} {\overset{•}{U}}_{A}=\frac{\sqrt{2}V_{DC}}{\pi }e^{j0},\;\;\;\;\;\;{\overset{•}{U}}_{B}=\frac{\sqrt{2}V_{DC}}{\pi }e^{-j\alpha },\;\;\;\;\;{\overset{•}{U}}_{C}=\frac{\sqrt{2}V_{DC}}{\pi }e^{-j\beta }\\ 0<\alpha ,\;\;\;\;\;\beta <\pi \end{array}\right.$$

**Figure 9:**
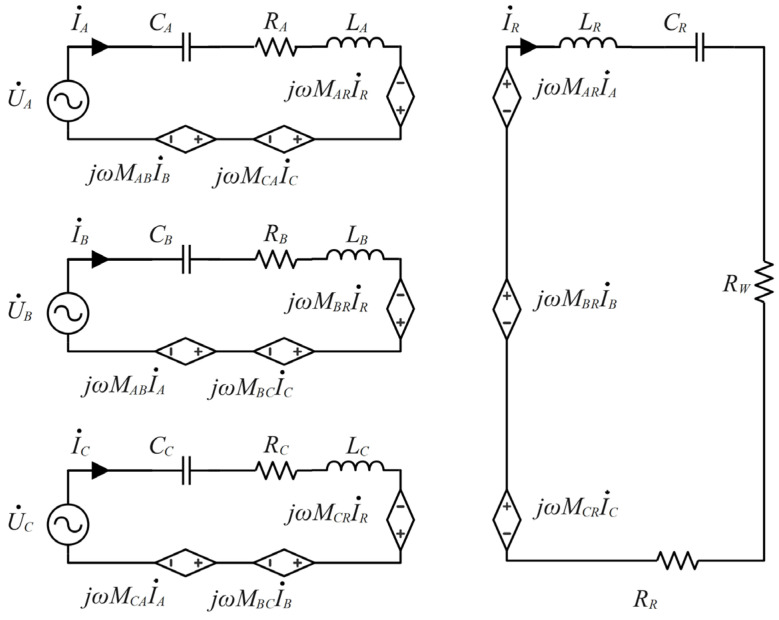
Equivalent decoupled circuit.

To simplify the analysis, the equivalent decoupled circuit model of the OWPT system is shown in Figure 9. Considering the mutual inductance $M_{AR}$, $M_{BR}$, and $M_{CR}$ of the transmitting and receiving coils, the KVL equation can be expressed as

(11) $$\left\{\begin{array}{l} \left[\begin{array}{c} {\overset{•}{U}}_{A}\\ {\overset{•}{U}}_{B}\\ {\overset{•}{U}}_{C} \end{array}\right]=\;\left[\begin{array}{ccc} Z_{A} & j\omega M_{AB} & j\omega M_{AC}\\ j\omega M_{AB} & Z_{B} & j\omega M_{BC}\\ j\omega M_{AC} & j\omega M_{BC} & Z_{C} \end{array}\right]\left[\begin{array}{c} {\overset{•}{I}}_{A}\\ {\overset{•}{I}}_{B}\\ {\overset{•}{I}}_{C} \end{array}\right]-\left[\begin{array}{c} j\omega M_{AR}\\ j\omega M_{BR}\\ j\omega M_{CR} \end{array}\right]\left[\begin{array}{c} {\overset{•}{I}}_{R} \end{array}\right]\\ \left[0\right]=\;\left[\begin{array}{ccc} j\omega M_{AR} & j\omega M_{BR} & j\omega M_{CR} \end{array}\right]\left[\begin{array}{c} {\overset{•}{I}}_{A}\\ {\overset{•}{I}}_{B}\\ {\overset{•}{I}}_{C} \end{array}\right]-\left[\begin{array}{c} Z_{R} \end{array}\right]\left[\begin{array}{c} {\overset{•}{I}}_{R} \end{array}\right] \end{array}\right.$$

Under the resonant state, the impedance can be further calculated as

(12) $$\left\{\begin{array}{l} Z_{A}={\left.R_{A}+j\omega L_{A}+\frac{1}{j\omega C_{A}}\right|}_{\omega =\omega_{0}}=R_{A}\\ Z_{B}={\left.R_{B}+j\omega L_{B}+\frac{1}{j\omega C_{B}}\right|}_{\omega =\omega_{0}}=R_{B}\\ Z_{C}={\left.R_{C}+j\omega L_{C}+\frac{1}{j\omega C_{C}}\right|}_{\omega =\omega_{0}}=R_{C}\\ Z_{R}={\left.R_{R}+R_{W}+j\omega L_{R}+\frac{1}{j\omega C_{R}}\right|}_{\omega =\omega_{0}}=R_{R}+R_{W} \end{array}\right.$$

The relationship between the equivalent load resistance $R_{W}$ and the actual load resistance $R_{L}$ is as follows:

(13) $$R_{W}=\frac{8}{\pi^{2}}R_{L}$$

Using COMSOL finite element simulation software to simulate the mutual inductance of the coil, the mutual inductance between HC1, SC2, and SC3 is in the order of magnitude of 10^−7^, which is negligible. Therefore, three-phase coils are spatially decoupled from each other, the mutual inductance $M_{AB}$, $M_{BC}$, and $M_{CA}$ can be equated to zero. Combined with (11) and (12), three-phase transmitting currents and receiving currents can be expressed as

(14) $$\left\{\begin{array}{l} \overset{•}{I_{A}}=\frac{\overset{•}{U_{A}}}{R_{A}+\frac{{\left(\omega_{0}M_{AR}\right)}^{2}}{R_{R}+R_{W}}},\;\;\;\;\;\;\overset{•}{I_{B}}=\frac{\overset{•}{U_{B}}}{R_{B}+\frac{{\left(\omega_{0}M_{BR}\right)}^{2}}{R_{R}+R_{W}}},\;\;\;\;\;\;\overset{•}{I_{C}}=\frac{\overset{•}{U_{C}}}{R_{C}+\frac{{\left(\omega_{0}M_{CR}\right)}^{2}}{R_{R}+R_{W}}}\\ \overset{•}{I_{R}}=\frac{j\omega \left(\sum_{X=A,B,C}M_{XR}\overset{•}{I_{X}}\right)}{R_{R}+R_{W}} \end{array}\right.$$

The system input power $P_{T}$, output power $P_{R}$, and efficiency η can be expressed as

(15) $$\left\{\begin{array}{c} P_{T}=V_{DC}\sum_{X=A,B,C}\left|\overset{•}{I_{X}}\right|\\ P_{R}={\left|\overset{•}{I_{R}}\right|}^{2}R_{W}\\ \eta =\frac{P_{R}}{P_{T}}=\frac{{\left|\overset{•}{I_{R}}\right|}^{2}R_{W}}{V_{DC}\sum_{X=A,B,C}\left|\overset{•}{I_{X}}\right|}\times 100\% \end{array}\right.$$

In fact, due to the differences in the dimensions of the transmitting and receiving structures, the coupling coefficients are so weak that $\frac{{\left(\omega_{0}M_{XR}\right)}^{2}}{R_{R}+R_{W}}\left(X=A,B,C\right)$ is negligible compared to $R_{X}\left(X=A,B,C\right)$. Therefore, the three-phase transmitting currents can be further simplified as

(16) $$\overset{•}{I_{A}}=\frac{\overset{•}{U_{A}}}{R_{A}},\;\;\;\;\;\;\overset{•}{I_{B}}=\frac{\overset{•}{U_{B}}}{R_{B}},\;\;\;\;\;\;\overset{•}{I_{C}}=\frac{\overset{•}{U_{C}}}{R_{C}}$$

Because of the consistency in the design of SC2 and SC3, $R_{B}$ is equal to $R_{C}$, the B-phase and C-phase currents are equal in amplitude and lag behind the A-phase currents by α and β, respectively. Clarifying this relationship lays a solid foundation for the subsequent analysis of spatial magnetic field vector synthesis.

### 4.2. Magnetic Field Vector Synthesis and Phase Difference Optimization

Based on the previous analysis of the magnetic field strength at the origin and the circuit analysis of the OWPT system, the synthetic magnetic field at the origin can be quantitatively analyzed. According to (7), the synthetic magnetic field H can be expressed as

(17) $$\left\{\begin{array}{l} H=A_{0}\sin(\omega_{0}t)k+B_{0}\sin(\omega_{0}t-\alpha )j+B_{0}\sin(\omega_{0}t-\beta )i\\ A_{0}={\left(\frac{4}{5}\right)}^{3/2}\frac{\sqrt{2}NV_{DC}}{\pi R_{A}r},\;\;\;\;\;B_{0}=\frac{\sqrt{2}NV_{DC}}{\pi^{2}RR_{B}}\sin\frac{\phi_{0}}{2}\cdot \left(\frac{8R^{2}h}{{\left(h^{2}+4R^{2}\right)}^{3/2}}+\frac{2h}{{\left(h^{2}+4R^{2}\right)}^{1/2}}\right) \end{array}\right.$$

where $A_{0}$ and $B_{0}$ represent the magnitude of the component of the magnetic field strength in each direction, from which the magnitude of synthetic magnetic field strength can be expressed as

(18) $$\left|H\right|=\sqrt{{A_{0}}^{2}\sin^{2}(\omega_{0}t)+{B_{0}}^{2}\left[\sin^{2}(\omega_{0}t-\alpha {)+\sin}^{2}(\omega_{0}t-\beta )\right]}$$

and the square of $\left|H\right|$ can be expressed as

(19) $$\begin{array}{ll} {\left|H\right|}^{2} & ={A_{0}}^{2}\sin^{2}(\omega_{0}t)+{B_{0}}^{2}\left[\sin^{2}(\omega_{0}t-\alpha {)+\sin}^{2}(\omega_{0}t-\beta )\right]\\ & ={B_{0}}^{2}\left(\sin^{2}\alpha +\sin^{2}\beta \right)+\left[{A_{0}}^{2}+{B_{0}}^{2}\left(\cos2\alpha +\cos2\beta \right)\right]\sin^{2}(\omega_{0}t)\\ & \;-{B_{0}}^{2}\left(\sin2\alpha +\sin2\beta \right)\sin(\omega_{0}t)\cos(\omega_{0}t) \end{array}$$

For the magnitude of the magnetic field strength to be constant, the coefficients before the time variable should be zero, leaving only the constant term.

(20) $$\left\{\begin{array}{l} {A_{0}}^{2}+{B_{0}}^{2}\left(\cos2\alpha +\cos2\beta \right)=0\\ {B_{0}}^{2}\left(\sin2\alpha +\sin2\beta \right)=0\\ {\left|H\right|}^{2}={B_{0}}^{2}\left(\sin^{2}\alpha +\sin^{2}\beta \right)\\ 0<\alpha ,\;\;\;\;\beta <180^{\circ } \end{array}\right.$$

Considering the symmetry of SC2 and SC3, it is useful to assume that α<β. From the first term of (20), the following can be obtained:

(21) $$\cos2\alpha =\pm \sqrt{1-\sin^{2}2\alpha },\;\;\;\;\;\cos2\beta =\pm \sqrt{1-\sin^{2}2\beta }$$

From the second term of (20), the following can be obtained:

(22) sin2α+sin2β=0⇒sin2α=−sin2β

Substituting (21) into the first term of (20), the following can be obtained:

(23) $${A_{0}}^{2}+{B_{0}}^{2}\left(\pm \sqrt{1-\sin^{2}2\alpha }\pm \sqrt{1-\sin^{2}2\alpha }\right)=0$$

As discussed in the following cases, where cos2α and cos2β have different signs or sin2α=1, (23) is reduced to ${A_{0}}^{2}=0$ and is thus excluded. The following discussion focuses on the case where cos2α and cos2β have the same sign.

(24) $$\left\{\begin{array}{l} {A_{0}}^{2}+{B_{0}}^{2}\left(\sqrt{1-\sin^{2}2\alpha }+\sqrt{1-\sin^{2}2\alpha }\right)=0\\ {A_{0}}^{2}+{B_{0}}^{2}\left(-\sqrt{1-\sin^{2}2\alpha }-\sqrt{1-\sin^{2}2\alpha }\right)=0\\ 45^{\circ }<\alpha <135^{\circ },\;where\;\alpha \neq 90^{\circ } \end{array}\right.$$

Since both $A_{0}$ and $B_{0}$ are greater than zero, the case where cos2α and cos2β have the same sign and both are greater than zero is ruled out; then, the only case left is where cos2α and cos2β have the same sign and both are less than zero, we can further derive the relationship between $A_{0}$, $B_{0}$, and α.

(25) $$\left\{\begin{array}{l} {A_{0}}^{2}+{B_{0}}^{2}\left(-\sqrt{1-\sin^{2}2\alpha }-\sqrt{1-\sin^{2}2\alpha }\right)=0\;\Rightarrow \;\frac{{A_{0}}^{2}}{{B_{0}}^{2}}=2\sqrt{1-\sin^{2}2\alpha }\\ 45^{\circ }<\alpha <135^{\circ },\;where\;\alpha \neq 90^{\circ } \end{array}\right.$$

The relationship between $A_{0}$ and $B_{0}$ needs to be satisfied by the nature of the sine function.

(26) $$\frac{{A_{0}}^{2}}{{B_{0}}^{2}}=2\sqrt{1-\sin^{2}2\alpha }\Rightarrow 0<A_{0}<\sqrt{2}B_{0}$$

The relationship between α and β can be deduced from (22).

(27) $$\alpha +\beta =180^{\circ },\;\;\;45^{\circ }<\alpha <\beta <135^{\circ },\;where\;\alpha ,\beta \neq 90^{\circ }$$

In summary, the theoretical phase difference α and β of the signal can be obtained from (26) and (27) under the condition that $0<A_{0}<\sqrt{2}B_{0}$. At this time, the magnetic field strength is constant as the direction changes continuously in a cycle. If the phase difference is $\alpha =\beta =0^{\circ }$, which is not optimized, then it is impossible to form a rotating magnetic field. The correctness of the idea was verified by a simulation and an experiment.

## 5. Simulation and Experimental Results

### 5.1. Proposed OWPT System

**Figure 10:**
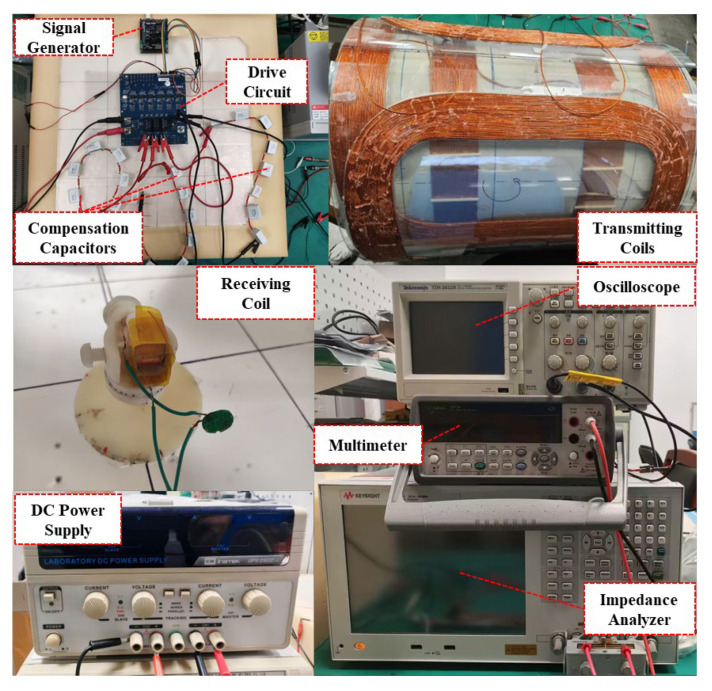
Prototype of the proposed OWPT system.

To verify the theoretical analyses above, a prototype of the OWPT system based on three-phase topology was implemented. As shown in Figure 10, HC1, SC2, and SC3 are sequentially wound on a cylindrical structure, with each set of coils isolated from one another by a thin plastic PVC hardboard. There is sufficient space within the internal column, allowing the patient to lie down comfortably to receive medical treatment. Table 1 provides the specifications for the OWPT system. The specific implementation details of the system are outlined below.

- Transmitting coils: The design specifications for the three coil pairs were developed based on Table 1, and the actual design specifications of SC2 and SC3 were verified using an impedance analyzer to ensure consistency.
- Receiving coil: As a PRC, the backstage delivers the rectified and voltage-stabilized output to the CR. A 30 Ω load is connected in series after the PRC and the compensation capacitors during the actual test to simulate the load of the CR during movement.
- Excitation signals: Based on the specifications in Table 1 and the experimental test, the values of α and β that are necessary for generating a rotating magnetic field during operation were calculated. The excitation signals were then adjusted using the signal generator.

### 5.2. Simulation and Experimental Verification

#### 5.2.1. Rotating Magnetic Field Simulation

**Figure 11:**
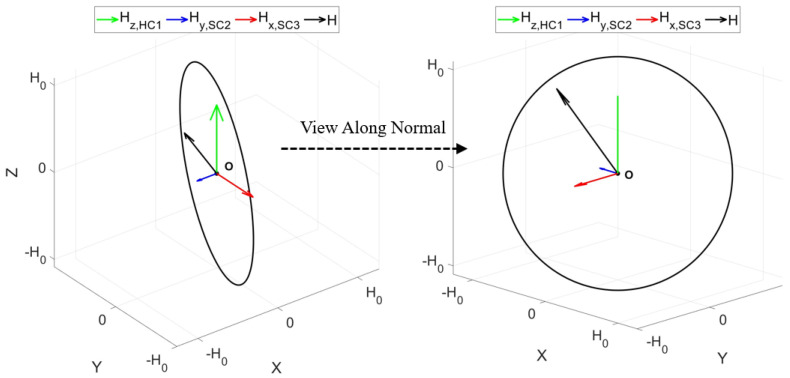
Magnetic field trajectory at the origin.

From (20), it is evident that the magnitude of the spatial synthetic magnetic field remains constant when the phase difference conditions are met. To further investigate its rotational trajectory, the spatial magnetic field was simulated and calculated using MATLAB. Theoretically, the three-dimensional coils generate a magnetic field strength ratio at the origin of approximately $A_{0}/B_{0}\approx 1.405$. When the three-dimensional coils operate together, the theoretical phase difference is $\alpha =85.378^{\circ }$ and $\beta =94.622^{\circ }$, respectively. The motion trajectory of the magnetic field over one working cycle is illustrated in Figure 11.

In Figure 11, $H_{0}=B_{0}\sqrt{\sin^{2}\alpha +\sin^{2}\beta }$ represents the magnitude of the magnetic field strength, where $B_{0}=\frac{\sqrt{2}NV_{DC}}{\pi^{2}RR_{B}}\sin\frac{\phi_{0}}{2}\cdot \left(\frac{8R^{2}h}{{\left(h^{2}+4R^{2}\right)}^{3/2}}+\frac{2h}{{\left(h^{2}+4R^{2}\right)}^{1/2}}\right)$. From Figure 11, it is clear that the magnetic field trajectory is circular, indicating that the magnitude of the magnetic field remains constant throughout one cycle while the direction sweeps around the circle. This implies that the magnetic field is uniform at each angle within the plane where the circular trajectory is centered. Generally, when the receiving coil experiences attitude shifts, the higher the uniformity of the magnetic field in its vicinity, the greater the stability of the received voltage.

**Figure 12:**
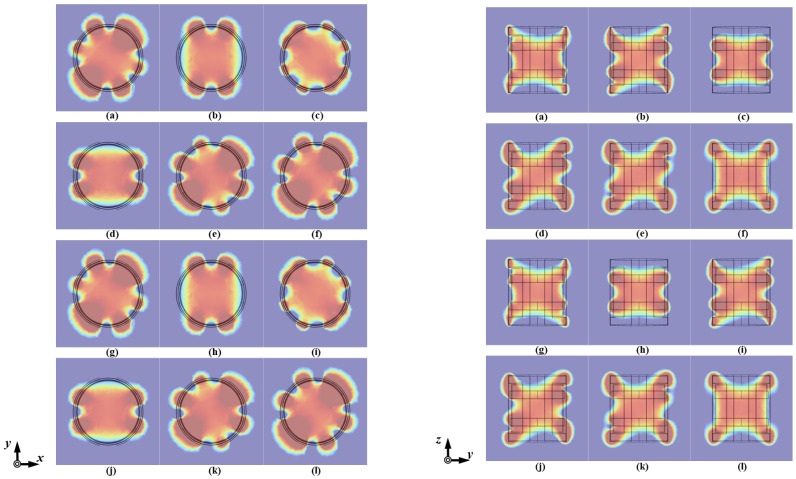
Magnetic field distribution of HC1 (**left**) and SC3 (**right**) at a different time instant. (**a**) $\omega t=\frac{\pi }{6}$. (**b**) $\omega t=\frac{\pi }{3}$. (**c**) $\omega t=\frac{\pi }{2}$. (**d**) $\omega t=\frac{2\pi }{3}$. (**e**) $\omega t=\frac{5\pi }{6}$. (**f**) ωt=π. (**g**) $\omega t=\frac{7\pi }{6}$. (**h**) $\omega t=\frac{4\pi }{3}$. (**i**) $\omega t=\frac{3\pi }{2}$. (**j**) $\omega t=\frac{5\pi }{3}$. (**k**) $\omega t=\frac{11\pi }{6}$. (**l**) ωt=2π.

To further visualize the omnidirectionality of the magnetic field, the OWPT system was simulated using the COMSOL finite element software, achieving an omnidirectional magnetic field through appropriate parameter settings. As shown in Figure 12, the magnetic field distribution over time is visualized from *xOy* and *yOz*, noting that the magnetic field distribution in the *xOz* and *yOz* planes exhibit symmetry. To emphasize the uniformity of the magnetic field strength distribution, the color rendering range was adjusted, clearly illustrating that while the magnetic field does not point in the same direction at different moments in a cycle, its strength remains stable and uniform across the spatial range.

#### 5.2.2. Results of the One-Dimensional Coil Experiment

**Figure 13:**
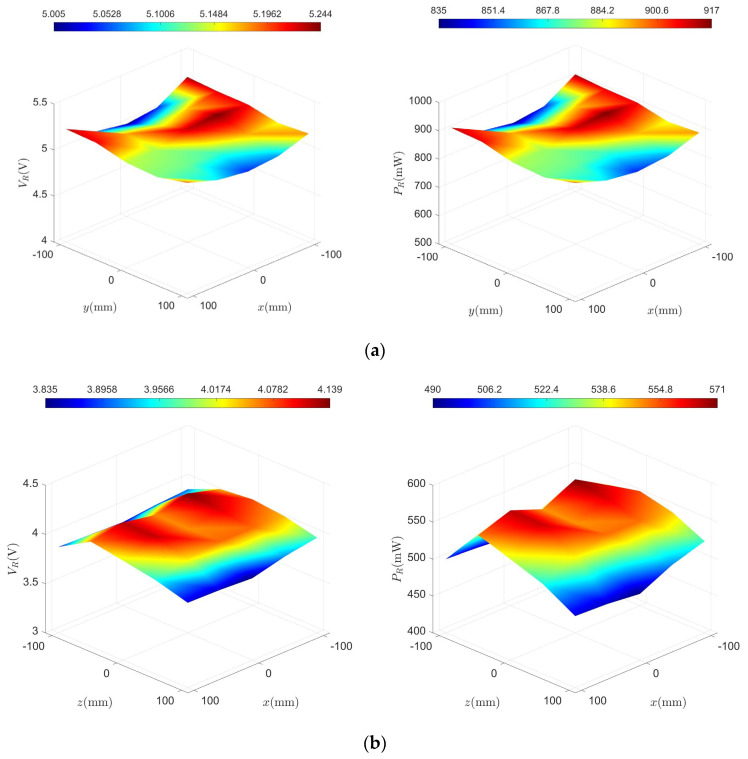

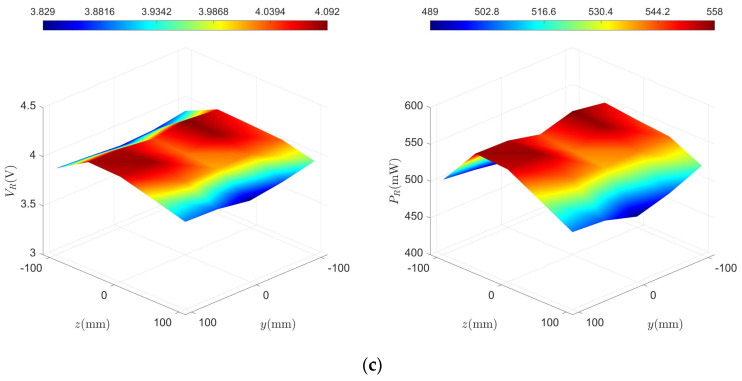
Spatial distribution of the received voltage and power of (**a**) HC1; (**b**) SC2; and (**c**) SC3.

**Figure 14:**
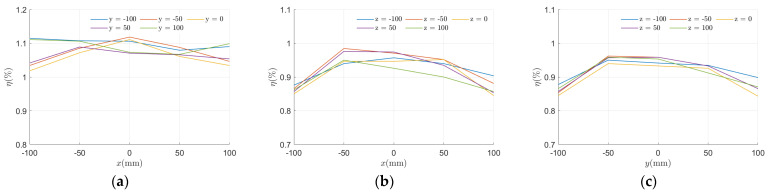
Distribution of power transfer efficiency at different locations of (**a**) HC1; (**b**) SC2; and (**c**) SC3.

To analyze the implementation effectiveness of the system prototype, power transfer tests were conducted for HC1, SC2, and SC3, with the receiving coils fixed to align with the x, y, and z axes, respectively. The distribution of the received voltage and power for each of the three coil pairs, tested individually, is shown in Figure 13. HC1 can provide a minimum received voltage of 5.005 V and a power transfer of 835 mW when powered individually. The experimental results for SC2 and SC3 are similar, ensuring a power supply of at least approximately 489 mW within the planar range. Figure 14a–c illustrates the distribution of power transfer efficiency. Among the three, HC1 achieved the highest average transmission efficiency at approximately 1.08%, followed by SC2 and SC3 at around 0.91%. This can be attributed to the relatively large distance between the transmitting and receiving coils, which leads to weaker coupling.

The statistical results of the transmission characteristics of the three groups of coils are shown in Table 2. Under the same operating voltage, HC1 exhibits the highest received voltage, while the power transfer characteristics of SC2 and SC3 are nearly identical. The average received voltage of HC1 is about 1.28 times that of SC2 and SC3. Moreover, the results demonstrate the smoothness of the received voltage over the planar range when the three sets of coils are powered individually. HC1 has the best uniformity (95.8%), followed by SC3, with SC2 having the lowest but still high uniformity (94.5%).

#### 5.2.3. Results of the Experiment on OWPT System Attitude Shifts 

**Figure 15:**
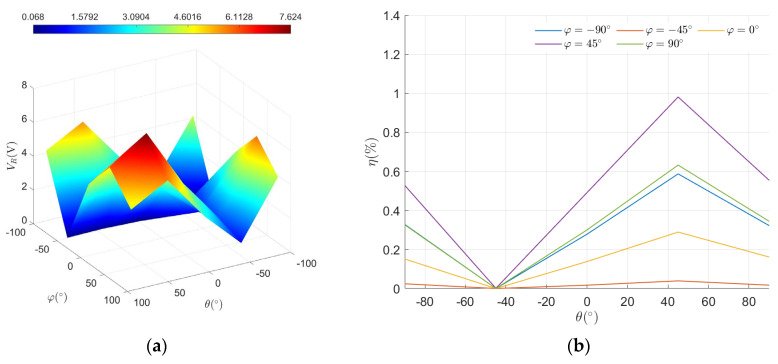
Received voltage (**a**) and power transfer efficiency (**b**) at different attitudes.

If the phase difference is not optimized, setting $\alpha =\beta =0^{\circ }$ makes the PRC highly sensitive to changes in attitude. Through calculations, it is theoretically determined that the optimal attitude for maximum PRC received voltage is $(\theta ,\;\varphi )=\left(47.57^{\circ },\;45^{\circ }\right)$, where θ is the angle between the normal of the PRC receiving plane and the *z*-axis, and φ is the angle between the normal of the PRC receiving plane and the *y*-axis. Figure 15 illustrates the actual received voltage and power transfer efficiency of PRC under different attitudes at the origin. The results confirm that, without optimizing the phase difference, the received voltage reaches a maximum value of 7.624 V at $(\theta ,\;\varphi )=\left(45^{\circ },\;45^{\circ }\right)$, corresponding to a power transfer efficiency of 0.98%, and a minimum received voltage of 0.068 V at $(\theta ,\;\varphi )=\left(-45^{\circ },\;-45^{\circ }\right)$, with a voltage fluctuation rate of up to 69.13%.

**Figure 16:**
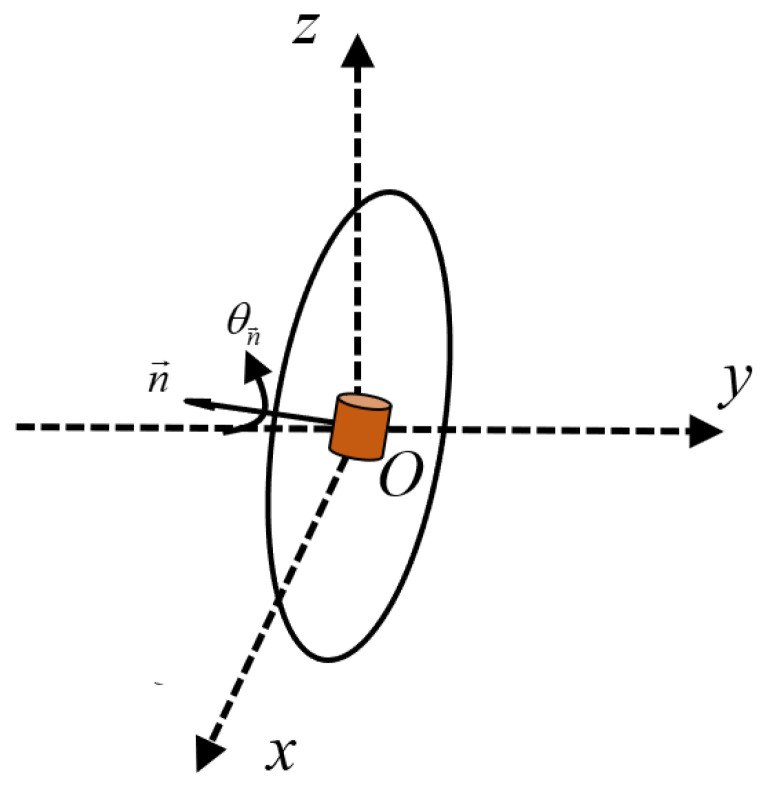
Schematic diagram of PRC rotation.

Based on the analysis in Section 4, when the specific condition $0<A_{0}<\sqrt{2}B_{0}$ is met, there exists values of α and β that make the spatially synthesized magnetic field a rotating magnetic field. Testing indicates that the three-dimensional coils generate a magnetic field strength ratio at the origin of approximately $A_{0}/B_{0}=1.288$. When the three-dimensional coils operate together, the practical phase difference is α=73.145° and β=106.855°, respectively. At this time, the unit normal vector of the synthetic magnetic field plane is $\vec{n}=\left(0.6751,-0.6751,0.2974\right)$, with an angle of 47.67° to both the *xOz* and *yOz* planes, closely matching the CR’s operating attitude within the intestine. The PRC is placed at the origin, with the normal vector of the receiving plane always being orthogonal to this normal vector, as shown in Figure 16. The PRC rotates about the origin along the axis of $\vec{n}$, with a rotation angle of $\theta_{\vec{n}}$. An experiment on attitude shifts was also conducted.

**Figure 17:**
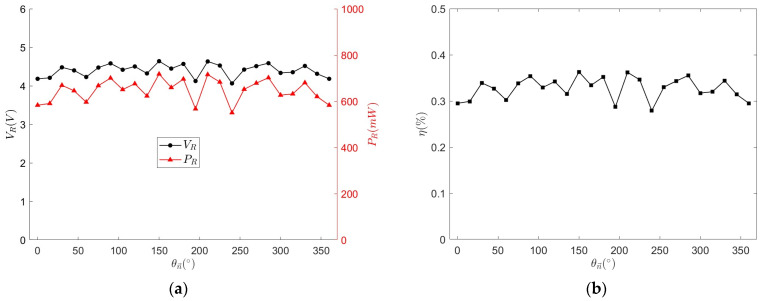
$V_{R},\;P_{R}$ (**a**) and η (**b**) at different $\theta_{\vec{n}}$.

The PRC is placed at the origin and $\theta_{\vec{n}}$ is varied from 0° to 360° in 15° increments. The variations in received voltage, power, and power transfer efficiency over one full rotation of the PRC are shown in Figure 17. Overall, the lowest received voltage is 4.063 V at $\theta_{\vec{n}}=240^{\circ }$, with a minimum received power of 550 mW. The average received voltage is 4.401 V, with an average power transfer efficiency of 0.33% and a voltage fluctuation rate of 7.68%, indicating good omnidirectionality of the rotating magnetic field in this plane, which is acceptable for the OWPT system.

## 6. Discussion and Conclusions

Wireless power transfer offers new opportunities for powering capsule robots, enabling stable detection and targeted drug delivery in medical applications. However, with an expanded workspace, positional and attitude shifts become critical factors that affect power transfer stability. In this article, an OWPT system based on the Helmholtz coil and saddle coil pairs is proposed for powering CRs. The parameters of the saddle coil pairs are optimized in terms of magnetic field uniformity and quality factor. The phase difference of excitation signals is optimized from the magnetic field vector synthesis point of view. Compared to the conventional WPT system, the system proposed in this article can provide a large range of uniform magnetic fields, and a two-dimensional omnidirectional magnetic field can be provided at the origin, which improves the position and attitude tolerance of the system. The experiments show that the three-dimensional coil possesses at least 94.5% uniformity and can receive at least 550 mW of power during the rotation at the origin, with a received voltage fluctuation rate of 7.68%.

Our study has achieved some progress in this area of research. However, there are still issues worth discussing and addressing. The proposed transmitting structure features a cylindrical design with three-dimensional coils wound around the cylinder, providing sufficient internal space to accommodate patients of varying body types for clinical diagnostic and therapeutic needs. However, the structure’s substantial size of 640 mm i3n diameter and 770 mm in height occupies considerable space, potentially affecting user mobility and comfort. To address this, several solutions have been proposed:

- Modular design: The transmitting structure could be reconfigured as a modular system, retaining only the basic cylindrical framework embedded within the examination table. The three-dimensional coils could be made detachable, allowing flexible installation and movement to suit different diagnostic and therapeutic scenarios.
- Platform optimization: The patient lying platform could be redesigned with added rollers and tracks, enabling smoother adjustments for different patient postures and improving overall usability.
- Coil design: Enhancing the coil design to improve magnetic field uniformity and intensity could minimize magnetic field leakage, making the transmitting structure more compact while maintaining adequate power supply.

For future applications, further research will focus on system integration, encapsulating the driving circuits and transmitting structure into a single unit. Additional mechanical features will also be incorporated to enable seamless mobility and internal adjustments, ensuring adaptability to more complex clinical diagnostic scenarios. Furthermore, external intestinal simulation experiments will be conducted to validate the system’s usability and performance in practical applications.

## Figures and Tables

**Table 1:** Specifications for the OWPT system.

Parameter	Value	Parameter	Value	Parameter	Value
$V_{DC}$	25 V	f	50 kHz	$L_{A}$	2.33 mH
$L_{B}$	3.13 mH	$L_{C}$	3.10 mH	$C_{A}$	4.35 nF
$C_{B}$	3.24 nF	$C_{C}$	3.27 nF	$R_{A}$	2.16 Ω
$R_{B}$	2.77 Ω	$R_{C}$	2.83 Ω	$L_{R}$	0.53 mH
$C_{R}$	19.30 nF	$R_{R}$	5.11 Ω	$R_{L}$	30 Ω
r	320 mm	h	770 mm	R	335 mm
$d_{r}$	15 mm	$d_{R}$	18.6 mm	$h_{R}$	14 mm
$\phi_{0}$	150°	$N_{A},\;N_{B},\;N_{C}$	30 × 2	$N_{R}$	120 × 3

**Table 2:** Statistical results of transmitting characteristics.

Coil	$V_{R\max}(V)$	$V_{R\min}(V)$	${\bar{V}}_{R}(V)$	${\bar{P}}_{R}(\mathrm{mW})$	*γ*(%)
HC1	5.244	5.005	5.148	883.4	95.8
SC2	4.139	3.835	3.991	530.9	94.5
SC3	4.092	3.829	3.981	528.3	95.0

## Data Availability

The data presented in this study are available upon request from the corresponding author. The data are not publicly available due to privacy restrictions.
